# Gut Microbiota Dysbiosis in BK Polyomavirus-Infected Renal Transplant Recipients: A Case-Control Study

**DOI:** 10.3389/fcimb.2022.860201

**Published:** 2022-05-27

**Authors:** Jian Zhang, Hao Qin, Mingyu Chang, Yang Yang, Jun Lin

**Affiliations:** ^1^ Department of Urology, Beijing Friendship Hospital, Capital Medical University, Beijing, China; ^2^ Beijing Key Laboratory of Tolerance Induction and Organ Protection in Transplantation, Beijing, China

**Keywords:** gut microbiota, BK polyomavirus, infection, renal transplantation, microbial dysbiosis

## Abstract

**Background:**

BK polyomavirus infection results in renal allograft dysfunction, and it is important to find methods of prediction and treatment. As a regulator of host immunity, changes in the gut microbiota are associated with a variety of infections. However, the correlation between microbiota dysbiosis and posttransplant BK polyomavirus infection was rarely studied. Thus, this study aimed to characterize the gut microbiota in BK polyomavirus-infected renal transplant recipients in order to explore the biomarkers that might be potential therapeutic targets and establish a prediction model for posttransplant BK polyomavirus infection based on the gut microbiota.

**Methods:**

We compared the gut microbial communities of 25 BK polyomavirus-infected renal transplant recipients with 23 characteristic-matched controls, applying the 16S ribosomal RNA gene amplicon sequencing technique.

**Results:**

At the phylum level, *Firmicutes*/*Bacteroidetes* ratio significantly increased in the BK polyomavirus group. *Bacteroidetes* was positively correlated with CD4/CD8 ratio. In the top 20 dominant genera, *Romboutsia* and *Roseburia* exhibited a significant difference between the two groups. No significant difference was observed in microbial alpha diversity. Beta diversity revealed a significant difference between the two groups. Nine distinguishing bacterial taxa were discovered between the two groups. We established a random forest model using genus taxa to predict BK polyomavirus infectious status, which achieved the best accuracy (80.71%) with an area under the curve of 0.82. Two genera were included in the best model, which were *Romboutsia* and *Actinomyces*.

**Conclusions:**

BK polyomavirus-infected patients had gut microbiota dysbiosis in which the *Firmicutes*/*Bacteroidetes* ratio increased in the course of the viral infection. Nine distinguishing bacterial taxa might be potential biomarkers of BK polyomavirus infection. The random forest model achieved an accuracy of 80.71% in predicting the BKV infectious status, with *Romboutsia* and *Actinomyces* included.

## Introduction

BK polyomavirus (BKV) infection is one of the most common but intractable complications following renal transplantation, which can result in renal allograft dysfunction and graft loss (Hariharan, 2006; Yi et al., 2017). Due to the existence of a buffering period from the onset of asymptomatic BKV infection to BKV-associated nephropathy, early determination of BKV infection and subsequent intervention are important for preventing the progression to BKV-associated nephropathy. Nowadays, detection of BKV almost depends on polymerase chain reaction (PCR), which is four times more sensitive than urine cytology for monitoring asymptomatic viruria, nonetheless lacking other predictive methods (Dalianis et al., 2019; Zakaria et al., 2019). Moreover, there have been no direct antiviral agents for BKV infection. Progressive reduction of immunosuppression according to the BKV viral load in the urine/blood samples or allograft biopsy results is the accepted therapeutic regimen (Hariharan, 2006; Dalianis et al., 2019; Myint et al., 2022). However, that may risk acute rejection, which challenges the treatment of BKV infection and leads to worse allograft survival (Baek et al., 2018). Thus, exploration of a new predictive method and treatment without the reduction of immunosuppression is needed.

Both innate immunity and adaptive immunity have been elucidated to play vital roles in controlling BKV infection (Ambalathingal et al., 2017). Recently, the gut microbiota, which is a complex ecosystem, has been demonstrated to be an important regulator of host immunity. Evidence suggests that gut microbial metabolites contribute to the development of both T-regulatory cells (Tregs) and the anti-inflammatory immune state. Especially the short-chain fatty acids, which are fermentation products of dietary fiber and carbohydrates by gut bacteria (Cummings, 1983), promote the conversion of naive CD4+ T lymphocytes to Tregs, contributing to immune suppression (Arpaia et al., 2013; Haase et al., 2018). Meanwhile, the metabolites can trigger immune responses against pathogens by inducing the secretion of pro-inflammatory cytokines (Kalina et al., 2002; Hosseinkhani et al., 2021). In brief, gut microbial metabolites are integral to immune homeostasis. Therefore, the dysbiosis of the gut microbiota may induce immune deficiency and subsequent infections by microbial metabolites. Several studies have demonstrated the correlation between gut microbiota dysbiosis and infections (Chan et al., 2019; Lee et al., 2019; Campisciano et al., 2020). In addition, bidirectional interactions between infections and the gut microbiota are reported that viral infections can also change the gut microbiota (Hanada et al., 2018). To date, the correlation between gut microbiota dysbiosis and posttransplant BKV infection is still unknown. Thus, this study aimed to characterize the gut microbiota in BKV-infected renal transplant recipients, compared with the controls, in order to explore the biomarkers that might be potential therapeutic targets and establish a prediction model for posttransplant BKV infection based on the gut microbiota.

## Materials and Methods

### Patient Cohort

Patients who received a first renal transplant within 2 years and received regular surveillance for BKV were included in the study. Exclusion criteria included the following: 1) multiorgan or pediatric transplants; 2) lacking BKV detection; 3) taking high-dose antibiotics; 4) delayed graft function early after renal transplantation; 5) concurrent human immunodeficiency virus, hepatitis virus, or *Mycobacterium tuberculosis* infection; 6) obese patients. BKV viral load was detected by PCR in all participants. All of them received low-dose sulfamethoxazole/trimethoprim and ganciclovir for prophylaxis after transplantation. Information about posttransplant infections, rejection, and medications was collected. A total of 48 recipients were enrolled and divided into the BKV group (n = 25) and the control group (n = 23), with a ratio of proximately 1/1. Fecal samples were collected after the diagnosis of BKV infection. The study was approved by the ethics committee of Beijing Friendship Hospital (2020-P2-212-01).

### Sample Collection

Fecal samples were collected by Faeces container (Sarstedt, Germany) without preserving reagent and stored at -80°C, and processed within 2 weeks after collection.

### Extraction of Genome DNA

Genome DNA was extracted from the samples using cetyltrimethylammonium bromide/sodium dodecyl sulfate (CTAB/SDS) method. DNA concentration and purity were monitored on 1% agarose gels. Extracted DNA was diluted to a concentration of 1 ng/μl using sterile water.

### Amplicon Generation

In this study, 16S ribosomal RNA (rRNA) genes were amplified using the specific primer Bakt_341F (5′- CCTACGGGNGGCWGCAG-3′) and Bakt_805R (5′-GACTACHVGGGTATCTAATCC-3′) (Herlemann et al., 2011). PCR reactions were performed in 30-μl reactions, containing 15 μl of Phusion^®^ High-Fidelity PCR Master Mix (New England Biolabs), 0.2 μl of forward and reverse primers, and 10 ng of template DNA. The thermal cycling protocol included initial denaturation at 95°C for 3 min, 25 cycles of denaturation at 95°C for 30 s, annealing at 55°C for 30 s, elongation at 72°C for 30 s, and finally 16°C for 2 min.

### PCR Product Quantification and Qualification

PCR products were mixed with an equal volume of 1× loading buffer (containing SYB green). Electrophoresis was performed on 2% agarose gel. Samples with a bright main strip around 460 bp (V3+V4) were chosen for further experiments (Vasileiadis et al., 2012).

### PCR Product Mixing and Purification

Since we mixed the PCR products in equi-density ratios, the mixture was purified using GeneJET Gel Extraction Kit (Thermo Scientific).

### Library Preparation and Sequencing

NEB Next Ultra DNA Library Prep Kit for Illumina (NEB, USA) was used to generate sequencing libraries according to recommendations from the manufacturer, and index codes were added. The library quality was assessed on the Qubit@ 2.0 Fluorometer (Life Technologies, CA, USA) and Agilent Bioanalyzer 2100 system. Finally, libraries were sequenced on Illumina MiSeq platform, and 250-bp paired-end reads were generated.

### Viral Infection Monitoring and Definition

The BKV infection status of each subject was confirmed by electronic medical records. Detection of BKV was performed by PCR (McNees et al., 2005). DNA was extracted from 200 µl of urine sample using the viral DNA/RNA extraction and purification kit (Xi’an Tianlong Science and Technology, Xi`an, China) according to the manufacturer’s instructions. PCR was performed using the ABI 7500 FAST Real-Time PCR System following the manufacturer’s recommendations and PCR fluorescence probe method with BKV nucleic acid detection kit (Beijing SinoMDgene Technology, Beijing, China). PCR reactions were performed in 25-μl reactions, containing 20-µl master mix and 5-µl template DNA. The thermal cycling protocol included initial denaturation at 95°C for 3 min, 40 cycles of denaturation at 94°C for 15 s, annealing, elongation, and signal acquisition at 60°C for 35 s, and finally 25°C for 1 min. BK viruria was defined as a positive result above the detectable level that the lower limit was 2.0E+03 copies/ml.

### Disease Prediction Model

We used the random Forest (version 4.6) package of R language to build the random forest model. The sequential forward selection method was used to select the best feature set. We started with the best feature with the largest classification accurate for feature selection, and then the other feature was added one by one. Each time the model classification accuracy was evaluated, the feature with the highest accuracy was added to the model. This process was repeated until the highest accuracy for the model was achieved. The 5-fold cross-validation was used to split the data into training and test datasets in model evaluation, and the cross-validation was repeated 20 times.

### Statistical Analysis

Alpha diversity and beta diversity on Bray–Curtis were measured using default parameters and QIIME2 tools. Principal coordinate analysis (PCoA) was generated to reveal the divergence between groups, and analysis of similarities (ANOSIM) was applied to test the significance of the clustering based on a distance matrix of Bray–Curtis. Linear discriminant analysis (LDA) Effect Size (LEfSe) was used to identify the distinguishing bacterial taxa between groups, limiting the log LDA score >4.0.

We used SPSS (ver. 26.0, SPSS Inc., Chicago, IL, USA) for statistical analysis. Measurement data were expressed as mean [standard deviation (SD)]. Student’s t-test and chi-square test were applied to compare the quantitative variables and the categorical variables between groups, respectively. Wilcoxon rank-sum test was used to compare the abundance of microbial species between groups. Spearman correlation analysis was applied to explore the correlation between the microbiota and the CD4/CD8 ratio. A *p* < 0.05 was considered to be statistically significant.

## Results

### Clinical Characteristics of Participants in the Study

The clinical characteristics of the BKV group and the control group were displayed in **Table 1**. There was no significant difference in gender, age, body mass index, concurrent diabetes, medications, concurrent infections, or clinical rejection between the two groups. The BKV viral load in urine was 2.35E+10 ± 9.98E+10 copies/ml in the BKV group. The CD4/CD8 ratio was 1.18 ± 0.71 in the BKV group, lower than that in the control group (*p* = 0.012).

**Table 1:** The clinical characteristics of the BKV group and the control group.

Parameters	BKV group (n = 25)	Control group (n = 23)	*p* value
Male (n, %)	12, 48	14, 61	0.371
Age (years)	44 ± 13	40 ± 11	0.209
BMI (kg/m^2^)	21.61 ± 3.39	22.37 ± 3.67	0.457
Diabetes (n)	4	2	0.743
Sulfamethoxazole/trimethoprim exposure (n)	20	22	0.230
Ganciclovir exposure (n)	15	18	0.173
Immunosuppressants (n)			
Tac	20	20	0.796
CsA	5	3	0.796
MPA	19	22	0.129
MZR	6	1	0.129
RPM	3	1	0.663
Concurrent infections (n)			
CMV	1	0	1.000
HPV-B19	1	1	1.000
VZV	0	1	0.479
Clinical rejection (n)	3	1	0.663
BKV viral load (copies/ml)	2.35E+10 ± 9.98E+10	ND	
CD4/CD8 ratio	1.18 ± 0.71	1.76 ± 0.81	0.012

BMI, body mass index; Tac, tacrolimus; CsA, cyclosporine A; MPA, mycophenolic acid; MZR, mizoribine; RPM, rapamycin; CMV, cytomegalovirus; HPV, human parvovirus; VZV, varicella-zoster virus; BKV, BK polyomavirus; ND, not detected.

### Sample Processing and Sequencing Results

A total of 48 fecal samples were processed for 16S rRNA gene amplicon sequencing. On average, 69,683 ± 6,297 sequence reads were generated per sample in the BKV group, and 68,779 ± 6,696 sequence reads were generated per sample in the control group. There was no significant difference between the two groups (*p* = 0.632).

### Microbial Community Structure

On average, the samples in the BKV group were characterized by comparable operational taxonomic unit (OTU) counts (mean: 218 ± 61 OTUs) compared with those in the control group (185 ± 70 OTUs), *p* = 0.083. There were a total of 390 shared OTUs between the two groups and 9 unique OTUs in the BKV group.

**Figure 1:**
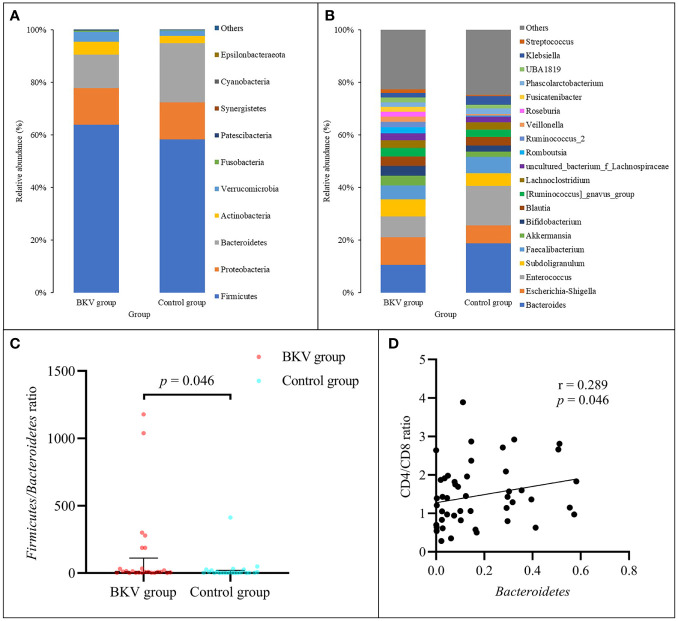
Abundance of bacterial taxa between the BKV group and the control group. **(A)** Top 10 dominant phyla. **(B)** Top 20 dominant genera. **(C)** The *Firmicutes*/*Bacteroidetes* ratio was significantly higher in the BKV group than that in the control group. **(D)** Spearman correlation analysis showed that *Bacteroidetes* was positively correlated with the CD4/CD8 ratio.

At the phylum level (**Figure 1A**), the abundance of *Firmicutes*, *Bacteroidetes*, *Proteobacteria*, *Actinobacteria*, and *Verrucomicrobia* accounted for over 99% in both groups. *Firmicutes*, *Actinobacteria*, and *Verrucomicrobia* were richer in the BKV group than those in the control group (63.92% vs. 58.32%, 5.08% vs. 2.69%, 3.59% vs. 2.30%), but there was no statistically significant difference (*p* = 0.458, *p* = 0.214, *p* = 0.469, respectively). *Bacteroidetes* and *Proteobacteria* were poorer in the BKV group than those in the control group (12.04% vs. 21.52%, 14.58% vs. 14.85%), but there was no statistically significant difference (*p* = 0.225 and *p* = 0.360, respectively). However, the *Firmicutes*/*Bacteroidetes* ratio was significantly higher in the BKV group than that in the control group (133.74 ± 306.79 vs. 26.65 ± 84.96, *p* = 0.046) (**Figure 1C**). The Spearman correlation analysis showed that *Bacteroidetes* was positively correlated with the CD4/CD8 ratio (r = 0.289, *p* = 0.046) (**Figure 1D**). In the top 20 dominant genera (**Figure 1B**), *Romboutsia* (2.26% in the BKV group vs. 0.16% in the control group, *p* = 0.022) and *Roseburia* (1.84% in the BKV group vs. 0.02% in the control group, *p* = 0.026) exhibited a significant difference between the two groups.

### Analysis of Microbial Diversity

**Figure 2:**
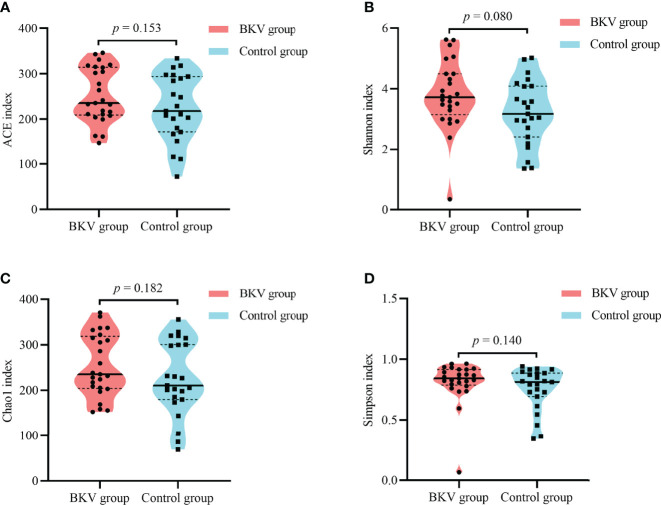
Alpha diversity indices between the BKV group and the control group. **(A)** ACE index. **(B)** Shannon index. **(C)** Chao1 index. **(D)** Simpson index. No significant difference was observed in microbial diversity. ACE, abundance-based coverage estimator.

As shown in **Table 2** and **Figure 2**, no significant difference was observed in the microbial alpha diversity.

**Table 2:** Microbial diversity in the BKV group and the control group.

Indexes	BKV group	Control group	*p* value
ACE	252.48 ± 60.29	224.53 ± 72.71	0.153
Shannon	3.80 ± 1.14	3.22 ± 1.07	0.080
Chao1	254.45 ± 68.02	225.27 ± 80.88	0.182
Simpson	0.81 ± 0.18	0.75 ± 0.18	0.140

The results were presented as mean ± SD for ACE index, Shannon index, Chao1 index, and Simpson index.

ACE, abundance-based coverage estimator.

### Clustering of Microbial Community

**Figure 3:**
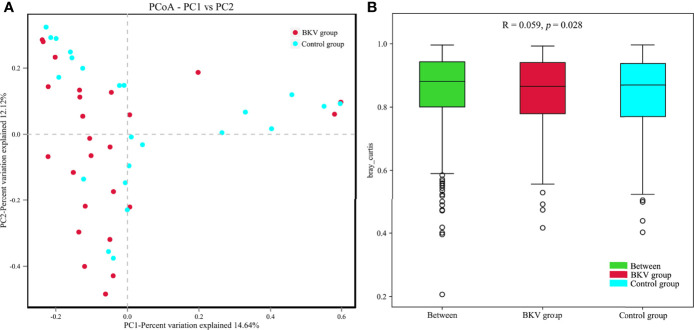
Comparative analysis between the BKV group and the control group. **(A)** PCoA was performed based on the Bray–Curtis distance. Clustering patterns of the BKV and control groups were identified by red and blue colors, respectively. PC1 and PC2 explained 14.64% and 12.12% of total variations, respectively. **(B)** ANOSIM at the OTU level was conducted. The difference in microbiota in the inter-group was bigger than that in the intra-group.

The PCoA plot was applied to evaluate the beta diversity based on Bray–Curtis distance analysis, which showed that the microbiota in the BKV group and the control group were clustered closely with some overlap (**Figure 3A**). The first principal component (PC1) and the second principal component (PC2) accounted for 14.64% and 12.12% of total variations, respectively. ANOSIM at the OTU level was conducted (**Figure 3B**). The R value was 0.059, while the *p* value was 0.028. The difference in microbiota in the inter-group was greater than that in the intra-group, indicating that the grouping was significantly meaningful.

### Difference in Bacterial Taxa Between the BKV Group and the Control Group

**Figure 4:**
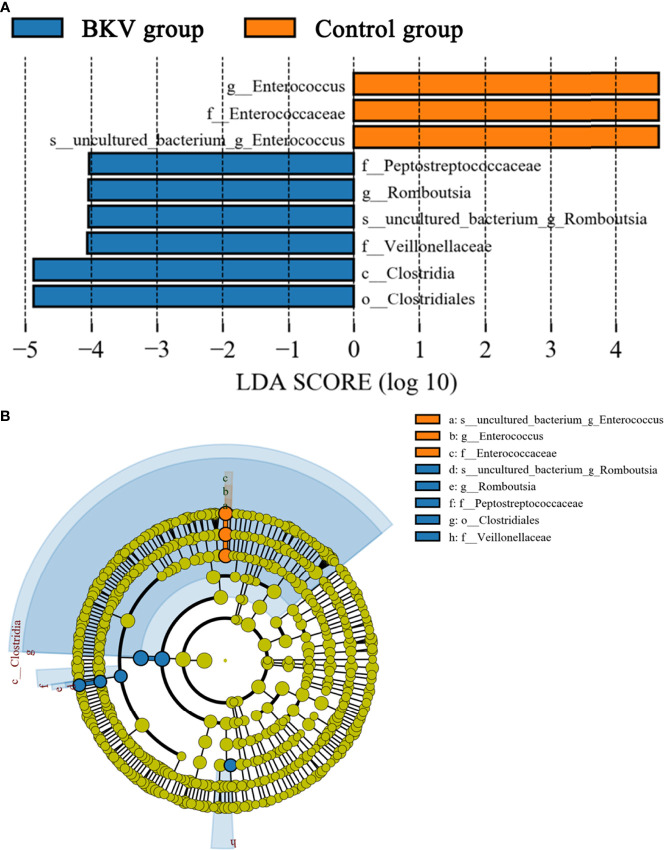
LEfSe analysis between the BKV group and the control group. **(A)** The LEfSe analysis demonstrated a significant difference in gut microbiota between the BKV group and the control group, with a log LDA score >4.0. The increase and decrease of bacterial taxa abundance in the BKV group were represented by blue and orange colors, respectively. **(B)** The cladogram demonstrated relationships among those taxa.

LEfSe was used to explore the biomarkers between the BKV group and the control group. We discovered 9 distinguishing bacterial taxa between the two groups, with a log LDA score >4.0. The abundance of the class *Clostridia*, order *Clostridiales*, family *Peptostreptococcaceae*, *Veillonellaceae*, genus *Romboutsia*, and species *uncultured bacterium of genus Romboutsia* was higher, while the abundance of family *Enterococcaceae*, genus *Enterococcus*, and species *uncultured bacterium of genus Enterococcus* was lower in the BKV group than that in the control group. The increase and decrease of bacterial taxa abundance in the BKV group were represented by different colors (**Figure 4**).

### Prediction Model of BKV Infection

**Figure 5:**
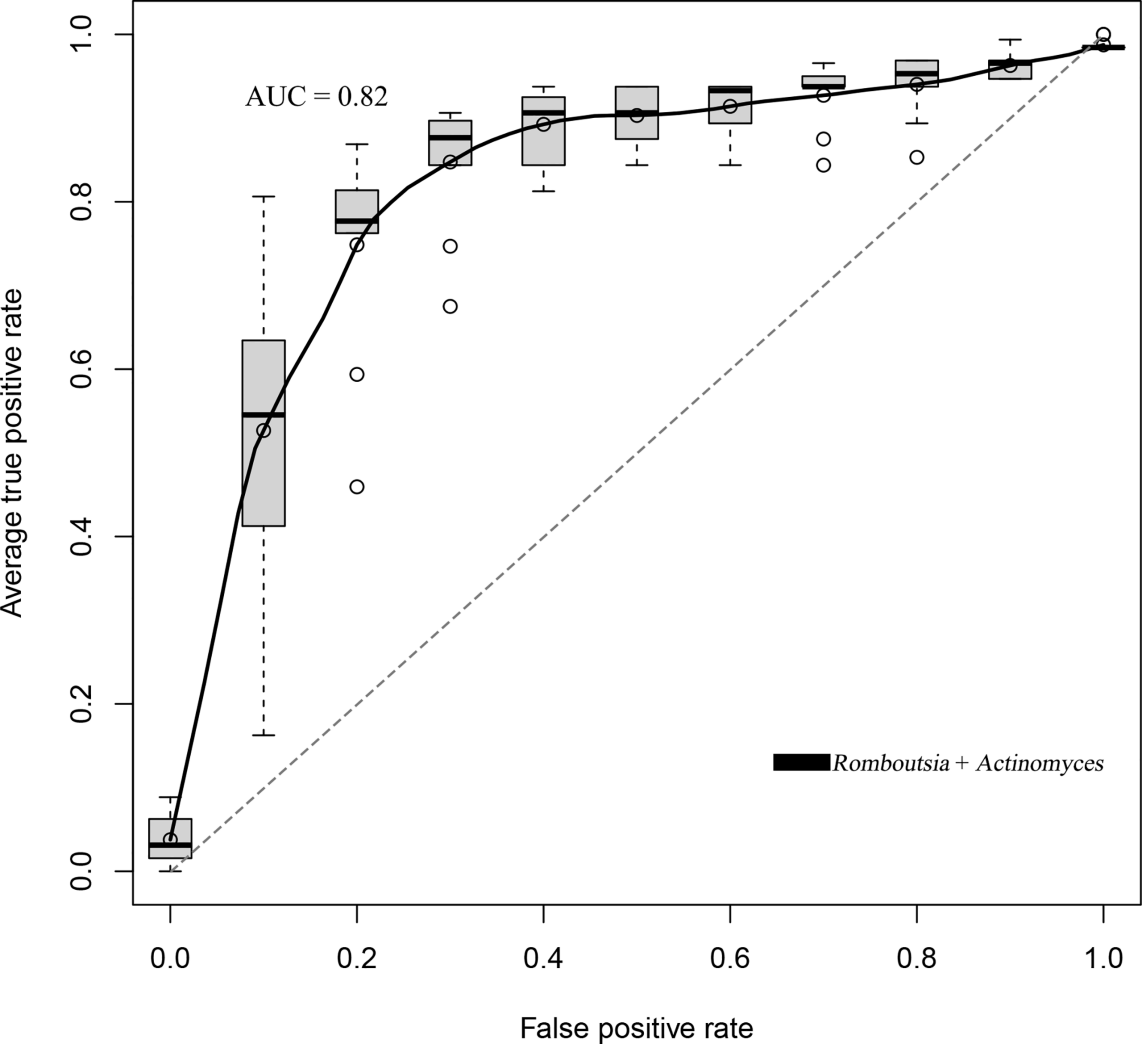
The ROC curve of the random forest model that trades off the rate of true positives against the rate of false positives. The best accuracy of the random forest model was 80.71% with an AUC of 0.82.

We established a random forest model using genus taxa to predict the BKV infectious status. The random forest model achieved the best accuracy (80.71%) to distinguish BKV-infected patients and those non-infected. The best model achieved an area under the curve (AUC) of 0.82 (**Figure 5**). Two genera were included in the best model, which were *Romboutsia* and *Actinomyces*.

## Discussion

The gut microbiota exhibited various physiological functions and plays a pivotal role in both the metabolism and immune system. A recent study demonstrated that the changes in the gut microbiota after renal transplantation might be associated with posttransplant infections (Lee et al., 2019). However, the correlation between microbiota dysbiosis and posttransplant BKV infection was rarely studied. Here, we discovered a gut microbiota dysbiosis in BKV-infected renal transplant recipients, which had not been reported previously.

In this study, the alpha diversity of the gut microbiota in BKV-infected patients was comparable to that in controls. However, the beta diversity revealed a significant difference between the two populations. At the phylum level, we found an obvious increase in the *Firmicutes*/*Bacteroidetes* ratio in the course of BKV infection. The phyla *Bacteroidetes* and *Firmicutes* contained the most abundant components of the human gut microbiota (Qin et al., 2010). Similarly, the elevated ratio had also been reported in several infectious diseases, including HBV infection (Zhu et al., 2019), HIV infection (González-Hernández et al., 2019), and *Clostridium difficile* infection (Bishara et al., 2013). Also, the alteration in this ratio was observed in obesity (Crovesy et al., 2020). An opposite situation was observed in some autoimmune diseases, such as type 1 diabetes (Zhou et al., 2020), Sjögren’s syndrome (Moon et al., 2020), and systemic lupus erythematosus (van der Meulen et al., 2019), in which the dysbiosis was characterized by a decrease in the *Firmicutes*/*Bacteroidetes* ratio. In the top 20 dominant genera, we found a significant increase in both *Romboutsia* and *Roseburia* in BKV-infected patients, which exhibited an anti-inflammatory effect and negatively correlated with inflammatory bowel disease (Schirmer et al., 2019; Qiu et al., 2020). Interestingly, we also found a lower CD4/CD8 ratio in BKV-infected patients, which indicated a higher risk of infection. Correlation analysis demonstrated that *Bacteroidetes* was positively correlated with CD4/CD8 ratio. Thus, this dysbiosis might contribute to BKV infection by immune modulation functions of the microbiota. However, it was still not known whether the microbial community was altered as a consequence of the infection process or the dysbiosis contributed to the infection.

Butyrate was a short-chain fatty acid, which was known to contribute to anti-inflammatory and immunosuppressive properties by affecting the differentiation, maturation, and function of dendritic cells and macrophages generated from human monocytes (Millard et al., 2002). It could induce extrathymic Treg differentiation, which was positively correlated with BK viremia (Arpaia et al., 2013; Furusawa et al., 2013; Karantanos et al., 2019). A recent study also demonstrated the impact of Tregs on immunity by Treg/IL-10/Th17 axis (Hui et al., 2019). In this study, we discovered 9 distinguishing bacterial taxa that were biomarkers of BKV infection that might be potential therapeutic targets. The abundance of the class *Clostridia*, order *Clostridiales*, family *Peptostreptococcaceae*, *Veillonellaceae*, genus *Romboutsia*, and species *uncultured bacterium of genus Romboutsia* was higher, while the abundance of family *Enterococcaceae*, genus *Enterococcus*, and species *uncultured bacterium of genus Enterococcus* was lower in the BKV group. Interestingly, the order *Clostridiales* (within the class *Clostridia*) includes many butyrate producers, such as the family *Peptostreptococcaceae*, *Veillonellaceae*, and genus *Romboutsia* (within the family *Peptostreptococcaceae*) (Esquivel-Elizondo et al., 2017; Liu et al., 2019; Chen et al., 2021), which might be correlated with BKV infection by their immunosuppressive properties. Inversely, the genus *Enterococcus* (within the family *Enterococcaceae*), a group of pro-inflammatory bacteria, declined in the feces of BKV-infected patients.

In this study, we established a random forest model to predict the BKV infectious status. We found that the inclusion of the genus taxa achieved the best accuracy (80.71%), which was better than that of other taxonomic categories of the gut microbiota. Moreover, only the combination of *Romboutsia* and *Actinomyces* achieved the best classification accuracy for the patients from the two groups. Either single feature or other feature combinations could not achieve a good classification effect for the model. We considered that this model could be clinically used as a non-invasive supplementary diagnostic method of BKV infection, and the efficiency could be improved with the continual enrollment of clinical samples in further studies.

## Conclusion

BKV-infected patients had a gut microbiota dysbiosis that the *Firmicutes*/*Bacteroidetes* ratio increased in the course of the viral infection. Nine distinguishing bacterial taxa might be potential biomarkers of BKV infection. The random forest model achieved an accuracy of 80.71% in predicting the BKV infectious status, with *Romboutsia* and *Actinomyces* included.

### Limitations

This study has limitations. Firstly, the sample size is limited. Secondly, the metagenome analysis is unused, which can provide more detailed information to explore the correlation between gut microbiota dysbiosis and BKV infection. Thirdly, a longitudinal study and metabolomics analysis are needed to explore and further understand the correlation between gut microbiota dysbiosis and the development of BKV infection.

## Data Availability Statement

The datasets presented in this study can be found in online repositories. The name of the repository and accession number can be found below: NCBI; PRJNA801362.

## Ethics Statement

This study was conducted according to the ethical guidelines of the Helsinki Declaration and approved by the ethics committee of Beijing Friendship Hospital. The patients/participants provided their written informed consent to participate in this study. None of the organs were procured from executed prisoners. All of the organs were procured after informed consent and allocated by the China Organ Transplant Response System.

## Author Contributions

JZ participated in design of the work, analysis and interpretation of data, drafting and revising the work. HQ and MC participated in acquisition and analysis of data and revising the work. YY participated in interpretation of data and revising the work. JL participated in conception and design of the work, interpretation of data, revising the work, acquisition of the funding, and supervision of the study. All authors contributed to the article and approved the submitted version.

## Funding

The study was funded by the Natural Science Foundation of Beijing Municipality (No. 7192043).

## Conflict of Interest

The authors declare that the research was conducted in the absence of any commercial or financial relationships that could be construed as a potential conflict of interest.

## Publisher’s Note

All claims expressed in this article are solely those of the authors and do not necessarily represent those of their affiliated organizations, or those of the publisher, the editors and the reviewers. Any product that may be evaluated in this article, or claim that may be made by its manufacturer, is not guaranteed or endorsed by the publisher.

## Abbreviations

ACE, abundance-based coverage estimator; ANOSIM, analysis of similarities; AUC, area under the curve; BKV, BK polyomavirus; LDA, linear discriminant analysis; LEfSe, linear discriminant analysis effect size; OTU, operational taxonomic unit; PCoA, principal coordinate analysis; PCR, polymerase chain reaction; rRNA, ribosomal RNA; SD, standard deviation; Tregs, T-regulatory cells.
